# Supplementary material for: Clay Nanotubes Loaded with Diazepam or Xylazine Permeate the Brain through Intranasal Administration in Mice
Source: Int J Mol Sci. 2023 Jun 2;24(11):9648. doi: 10.3390/ijms24119648 (PMC10253593; doi:10.3390/ijms24119648)

Supplemental Figures for

Clay Nanotubes Loaded with Diazepam or Xylazine Permeate the Brain through Intranasal Administration in Mice

Yaswanthi Yanamadala [1, †], Mahdi Y. Saleh [2,†], Afrika A. Williams [1] and Yuri Lvov [2,*], Teresa A. Murray [1,*]

1 Center for Biomedical Engineering and Rehabilitation Sciences, Louisiana Tech University, Rustom, LA 71270, USA; yashwanthi789@gmail.com (Y.Y.); afrika.williams@gmail.com (A.A.W.)

2 Institute for Micromanufacturing, Louisiana Tech University, Rustom, LA 71270, USA; mahdisaleh95@gmail.com

***** Correspondence: ylvov@latech.edu (Y.L.); tmurray@latech.edu (T.A.M.); Tel. : +1- (602)-692-3951

† These authors contributed equally to this work.

Figure S1. Representative images from mice sacrificed four hours after treatment with 3 mg/mL of HNT/RITC via intraperitoneal delivery. DAPI-stained nuclei (A, blue) and HNT/RITC (B, orange) in the same slice. Scale bar is 200 μm.

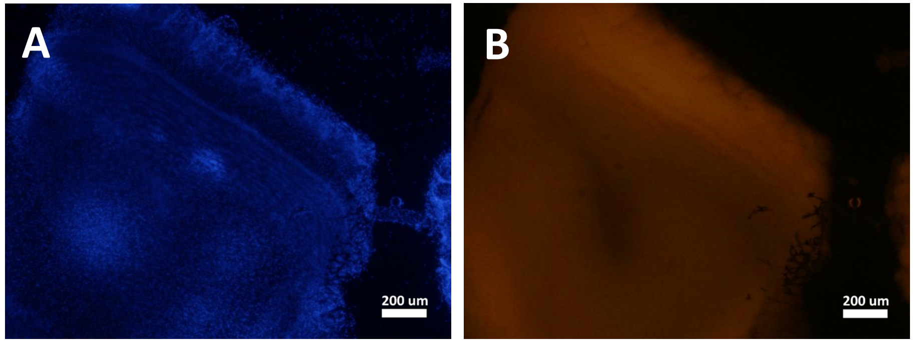

Figure S2. Representative images of the olfactory bulb from mice sacrificed at 4, 12, 24, and 48 hours after treatment with 3 mg/mL of HNT/RITC via intranasal (IN) delivery. DAPI-stained nuclei (left column, blue) and HNT/RITC (right column, orange) in the same slice. Scale bar is 200 µm for A-D and 100 µm for E-H.

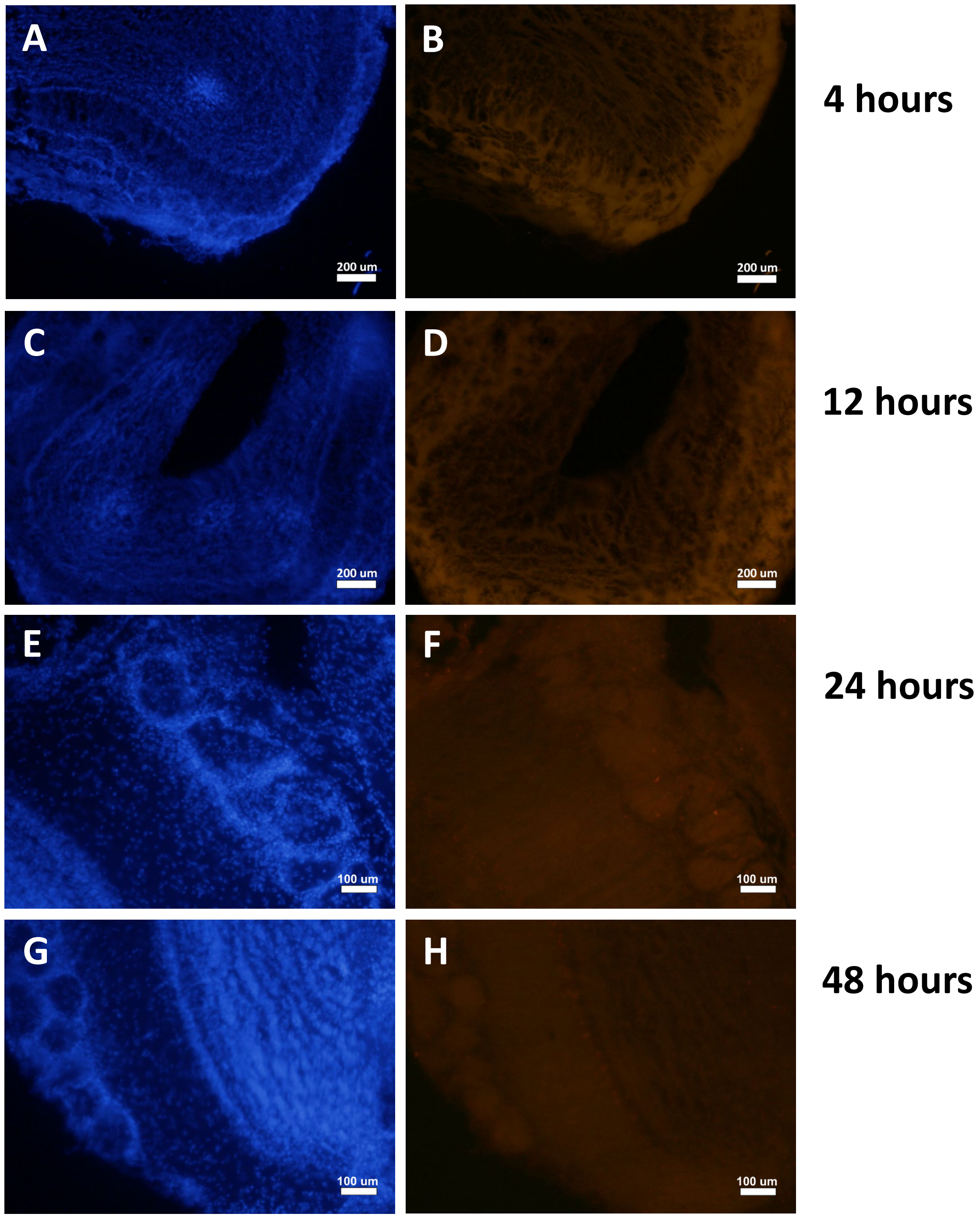

Figure S3. Representative images of the cortex from mice sacrificed at 4, 12, 24, and 48 hours after treatment with 3 mg/mL of HNT/RITC via IN delivery. DAPI-stained nuclei (left column, blue) and HNT/RITC (right column, orange) in the same slice. Scale bar is 200 µm for A-D and 100 µm for E-H.

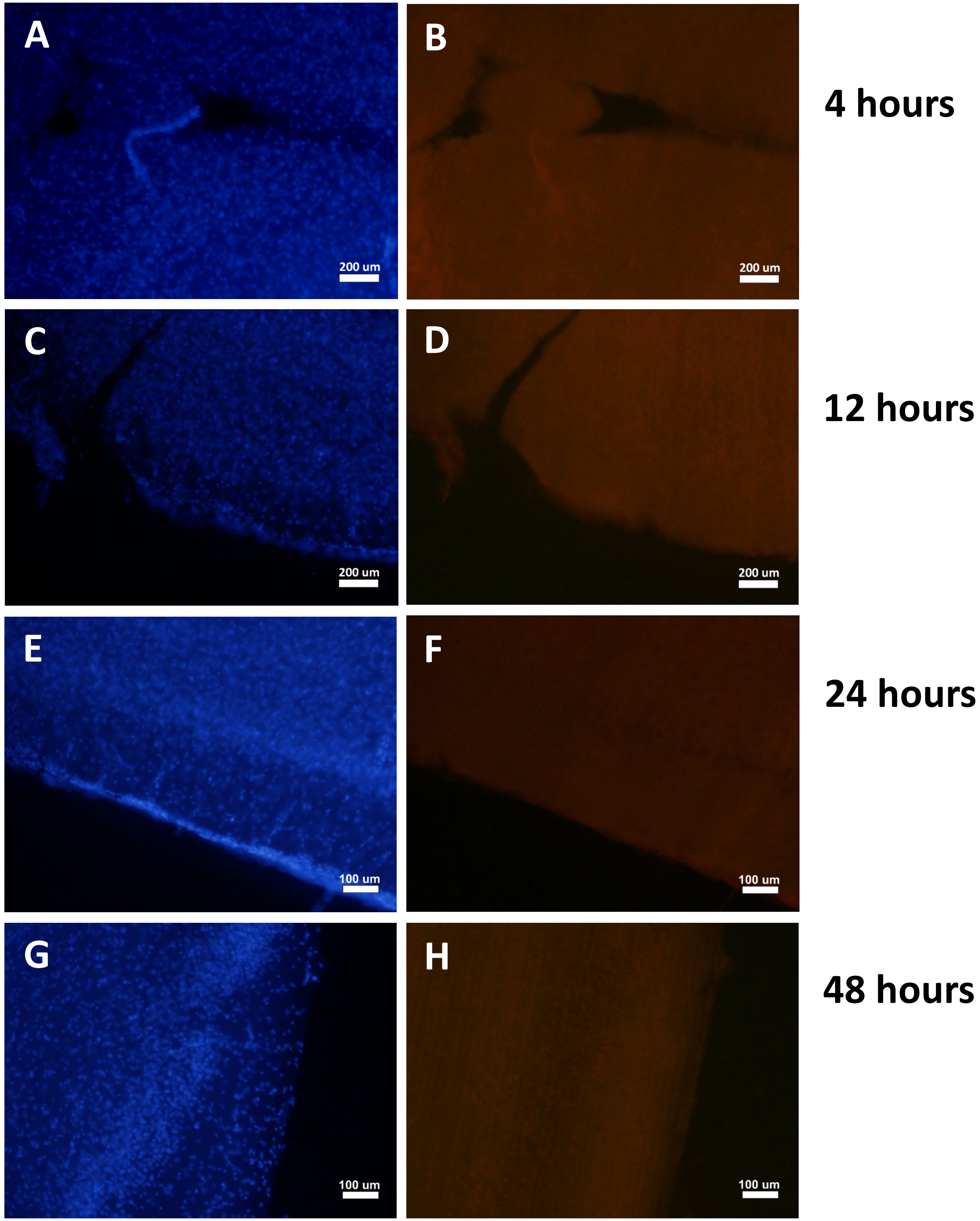

Figure S4. Representative images of the olfactory bulb (A-B) and the cortex (C-D) from mice sacrificed at 72 hours after treatment with 3 mg/mL of HNT/RITC via IN delivery. DAPI-stained nuclei (left column, blue) and HNT/RITC (right column, orange) in the same slice. No fluorescence from RITC was observed. Scale bar is 200 µm for A-B and 100 µm for C-D.

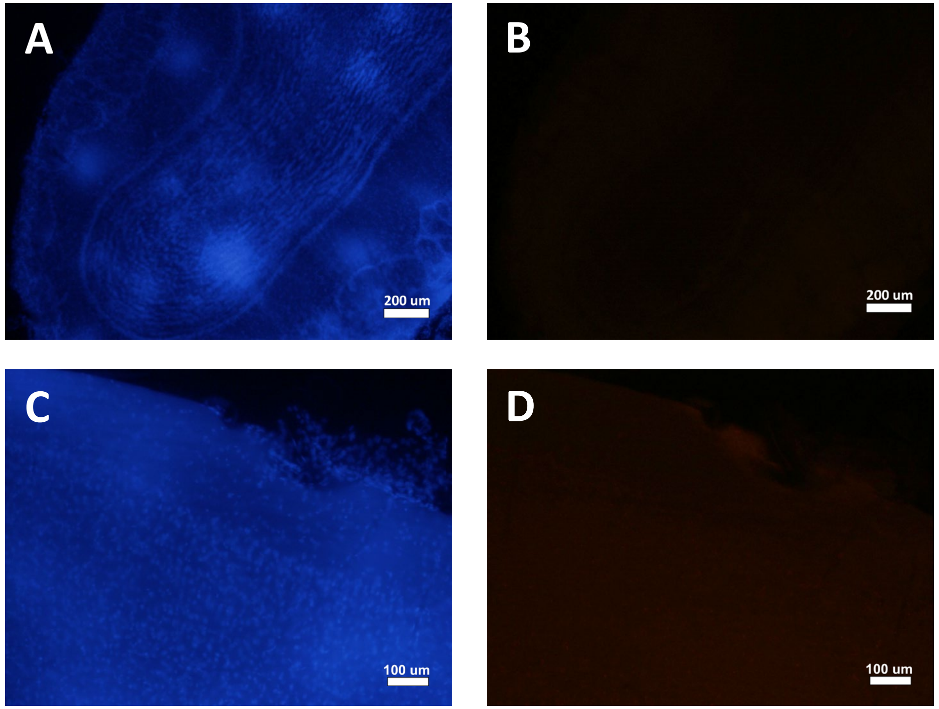

Supplement: Supplementary file 1 [file ijms-24-09648-s001.zip › ijms-2404918-supplementary.pdf]
